# Prolonged cycling reduces power output at the moderate-to-heavy intensity transition

**DOI:** 10.1007/s00421-022-05036-9

**Published:** 2022-09-20

**Authors:** Julian D. Stevenson, Andrew E. Kilding, Daniel J. Plews, Ed Maunder

**Affiliations:** Sports Performance Research Institute New Zealand, Auckland University of Technology, Auckland, New Zealand

**Keywords:** Cycling, Thresholds, Durability, Fatigue resistance, Exercise, Duration

## Abstract

**Purpose:**

To determine the effect of prolonged exercise on moderate-to-heavy intensity transition power output and heart rate.

**Methods:**

Fourteen endurance-trained cyclists and triathletes took part in the present investigation (13 males, 1 female, V·O_2_peak 59.9 ± 6.8 mL^.^kg^−1.^min^−1^). Following a characterisation trial, participants undertook a five-stage incremental step test to determine the power output and heart rate at the moderate-to-heavy intensity transition before and after two hours of cycling at 90% of the estimated power output at first ventilatory threshold (VT_1_).

**Results:**

Power output at the moderate-to-heavy intensity transition significantly decreased following acute prolonged exercise when determined using expired gases (VT_1_, 217 ± 42 W vs. 196 ± 42 W, *P* < 0.0001) and blood lactate concentrations (LoglogLT, 212 ± 47 W vs. 190 ± 47 W, *P* = 0.004). This was attributable to loss of efficiency (VT_1_, -8 ± 10 W; LoglogLT, − 7 ± 9 W) and rates of metabolic energy expenditure at the transition (VT_1_, − 14 ± 11 W; LoglogLT, − 15 ± 22 W). The heart rate associated with the moderate-to-heavy intensity transition increased following acute prolonged exercise (VT_1,_ 142 ± 9 beats^.^min^−1^ vs. 151 ± 12 beats^.^min^−1^, *P* < 0.001; LoglogLT, 140 ± 13 beats^.^min^−1^ vs. 150 ± 15 beats^.^min^−1^, *P* = 0.006).

**Conclusion:**

These results demonstrate the external work output at the moderate-to-heavy intensity transition decreases during prolonged exercise due to decreased efficiency and rates of metabolic energy expenditure, but the associated heart rate increases. Therefore, individual assessments of athlete ‘durability’ are warranted.

**Supplementary Information:**

The online version contains supplementary material available at 10.1007/s00421-022-05036-9.

## Introduction

Endurance athletes commonly perform physiological assessments for estimating the external work outputs that demarcate the boundaries between exercise intensity domains. These intensity domain transitions are used to assess performance capability, regulate training and competition intensities, monitor training load, and quantify adaptations to training (Maunder et al. 2021). The moderate and heavy exercise intensity domains are defined by physiological responses, including distinct blood lactate and whole-body oxygen uptake (V·O_2_) kinetics profiles (Burnley and Jones 2018; Jones et al. 2019).

An under-studied effect of prolonged exercise is the likely reduction in external work rates observed at the intensity transitions over time; the resilience to which we termed ‘durability’ (Maunder et al. 2021). A series of recent studies reported a reduction in the so-called critical power following prolonged heavy-intensity cycling, and indicated that the magnitude of the reduction was sensitive to carbohydrate availability (Clark et al. 2018, 2019a, b). Less is understood regarding the effects of acute prolonged exercise on the moderate-to-heavy intensity transition, which may plausibly also decline with prolonged exercise in a manner related to carbohydrate availability. Specifically, depletion of the intramyofibrillar glycogen store during prolonged exercise appears to impair muscle contractile function (Ørtenblad et al. 2013). Therefore, following prolonged exercise, intramyofibrillar glycogen depletion-induced impairment of specific muscle fibres may increase the mechanical and metabolic burden of a given power output on a smaller pool of fully functional fibres. Speculatively, this could increase the fibre-specific work rate at a given power output, reducing the power output achieved at the moderate-to-heavy intensity transition following prolonged exercise.

A reduction in gross cycling efficiency with prolonged moderate-intensity exercise has been observed (Passfield and Doust 2000; Hopker et al. 2017). This would theoretically contribute to reduced power output at intensity transitions following prolonged exercise, even if the rate of metabolic energy expenditure associated with the transitions is maintained over time. The contributions made by changes in gross cycling efficiency and rates of metabolic energy expenditure to prolonged exercise-induced changes in the power output at the moderate-to-heavy transition could be quantified by measurement of the energy expenditure vs. power output relationship, and identification of the rate of energy expenditure associated with the moderate-to-heavy intensity transition, before and after prolonged exercise.

Given the possibility that power outputs at intensity domain transitions may decrease during prolonged exercise, the use of data generated in well-rested physiological profiling assessments to regulate training intensity could result in inadvertent drift into higher intensity domains over time (Maunder et al. 2021). A common strategy employed by endurance athletes to combat this is the use of heart rates observed at intensity transitions to regulate training intensity. As progressive increases in heart rate at given power outputs can occur during prolonged exercise (Coyle and Gonzalez-Alonso 2001), an athlete using heart rate for training intensity regulation may progressively reduce their power output to remain at the target heart rate. Surprisingly, the degree to which cardiovascular drift during prolonged exercise reflects shifts in intensity domain transitions has not been established. Understanding the relationship between cardiovascular drift and acute exercise-induced changes in the power outputs at intensity domain transitions has implications for how heart rate monitoring might be used for training intensity regulation during prolonged exercise.

Therefore, the aims of the present investigation were to: (i) quantify the effects of prolonged cycling on the power output at the moderate-to-heavy intensity transition, (ii) determine the contributions made by changes in gross cycling efficiency and rates of metabolic energy expenditure at the moderate-to-heavy transition to prolonged cycling-induced changes in moderate-to-heavy intensity transition power output, and (iii) quantify the effects of prolonged cycling on the heart rate associated with the moderate-to-heavy intensity transition. We hypothesised that moderate-to-heavy transition power output would decrease following prolonged cycling due to decreased gross cycling efficiency and rates of metabolic energy expenditure at the transition, but that the heart rate associated with the transition would remain consistent over time.

## Methods

### Ethical approval

This study was performed in accordance with the standards of the Declaration of Helsinki, 2013. The Auckland University of Technology Ethics Committee approved all procedures (21/253), and all participants provided written informed consent prior to participation. This study was not registered in a database. Data associated with this study are available from the corresponding author upon reasonable request.

### Participants

Fourteen endurance-trained cyclists and triathletes took part in the present investigation (13 males, 1 female, age 34 ± 10, height 178.1 ± 5.6 cm, mass 71.2 ± 6 kg, V·O_2_peak 59.9 ± 6.8 mL^.^kg^−1.^min^−1^, training volume 9 ± 3 h^.^week^−1^). A priori sample size estimation indicated that a total sample size of 15 was required to detect a large magnitude (ES = 0.7) reduction in power output at the moderate-to-heavy intensity transition with 80% statistical power using the G*Power software package. A large magnitude effect size was used for this calculation based on previous studies showing the effect of prolonged exercise on the heavy-to-severe intensity transition (Clark et al. 2018, 2019a, b). A one-tailed test was used as it seemed implausible that the moderate-to-heavy intensity power output would increase following acute prolonged exercise. Data collection was interrupted by a COVID-19 lockdown and one participant dropped out. All participants were free of recent (< 3 months) musculoskeletal injury and chronic disease and habitually training > 5 h^.^week^−1^ in cycling-based endurance sports. This study was performed in accordance with the standards of the Declaration of Helsinki, 2013, and the Auckland University of Technology Ethics Committee approved all procedures (21/253).

### Study design

**Fig. 1:**
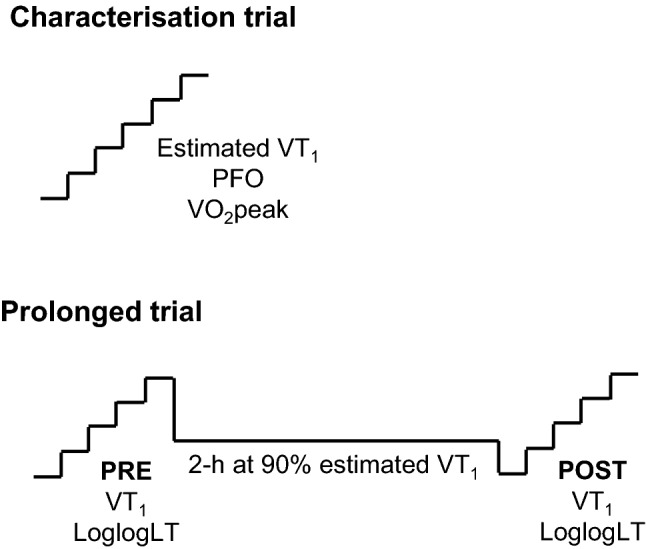
Schematic overview of the two laboratory visits

Participants visited the laboratory on two occasions, ~ 7 d apart, for: (i) a characterisation trial involving a maximal, incremental cycling test after an overnight fast and (ii) a prolonged trial, which involved a prolonged cycling assessment with estimation of the first ventilatory threshold (VT_1_) before and after two hours of moderate-intensity cycling, after an overnight fast (Fig. 1). The order of visits was not randomised as the incremental test data were used to define the parameters of the prolonged trial.

### Characterisation trial

Participants initially reported to the laboratory for an incremental cycling test. Participants arrived after a 10-h overnight fast having refrained from vigorous exercise for 24 h and having ingested ~ 1 L of plain water ~ 2 h beforehand. Height and body mass was first measured. Cycling subsequently commenced on an electromagnetically braked ergometer at 95 W, and the power output initially increased by 35 W every 3 min (Excalibur Sport, Lode BV, Groningen, NET). Expired gases were collected continuously using indirect calorimetry (TrueOne 2400, ParvoMedics, UT, USA). Heart rate was measured continuously using a chest-strap heart rate monitor (Tickr, Wahoo Fitness, Atlanta, USA).

Once the respiratory exchange ratio exceeded 1.0 and clear signs of increased V̇E^.^V·̇O_2_^−1^ emerged, power output was increased by 35 W every minute until task failure. The V·̇O_2_peak was identified as the highest 15-s averageV·̇O_2_, and VT_1_ was identified as the V·̇O_2_ at which a systematic rise in V̇E^.^V·̇O_2_^−1^ occurred. This V·̇O_2_ was converted to a power output by linear fit of the power output vs.V·̇O_2_ relationship, using the last minute of V·̇O_2_ data from each 3-min stage.

The last minute of expired gas data in each 3-min stage was used to determine whole-body fat oxidation rates through standard calculations (Jeukendrup and Wallis 2005) (Eq. 1). The highest observed rate of whole-body fat oxidation was accepted as the peak fat oxidation rate (PFO), as per our recent work (Maunder et al. 2022).

1 $${\text{Whole body fat oxidation rate }}(g \cdot \min^{ - 1} )\, = \,1.695\, \times \,\dot{V}O_{2} {-}1.701\, \times \,\dot{V}CO_{2}$$

where V·̇O_2_ and V̇CO_2_ are in L^.^min^−1^.

### Prolonged trial

Participants arrived for the prolonged trial after a 10-h overnight fast, having refrained from vigorous exercise for 24 h, and having ingested ~ 1 L of plain water ~ 2 h beforehand. Following measurement of body mass, the experimental trial commenced on the same electromagnetically braked ergometer as the initial assessment with a 5-min warm-up at 100 W, followed by a five-stage incremental assessment to determine the power output and heart rate at the moderate-to-heavy intensity transition. The incremental test began with 4-min at 50 W below the previously estimated VT_1_ power output, and power output increased by 25 W per increment.

Expired gases and heart rate were measured continuously (TrueOne 2400, ParvoMedics, UT, USA; Tickr, Wahoo Fitness, Atlanta, USA), and a fingerprick capillary blood sample was obtained in the last 30-s of each increment for measurement of blood lactate concentration using an automated analyser (Lactate Pro 2, Arkray). These data were used to quantify the power output and heart rate at the moderate-to-heavy intensity transition prior to prolonged exercise (PRE, see Data analysis section below). Participants then cycled for 5 min at 100 W, and then at 90% of the previously estimated power output at VT_1_ for 2 h. Heart rate was recorded throughout, and participants consumed plain water ad libitum. Expired gases were collected for 4 min, every 15 min.

Following the two-hour constant work-rate phase, participants again cycled for 5 min at 100 W before repeating the five-step incremental exercise assessment. These data were used to quantify the power output and heart rate at the moderate-heavy intensity transition following prolonged exercise (POST). Sweat loss was also assessed by measurement of pre- and post-exercise body mass, and water consumption. Total water consumption was recorded by measuring the mass of the bottle before and after use and refilling, and was then added to changes in body mass to calculate total sweat loss. If participants used the toilet during the trial, body mass was recorded before and after and accounted for in sweat loss calculations.

### Estimation of the moderate-to-heavy transition

The PRE and POST moderate-to-heavy intensity transitions were estimated using expired gas and blood lactate data. Specifically, using expired gas data, the moderate-to-heavy intensity transitions in the PRE and POST assessments of the experimental trial were estimated using the VT_1_ method in accordance with the procedures described above for the initial assessment. The V·̇O_2_ at VT_1_ was converted to a power output by linear fit of the power output vs. V·̇O_2_ relationship, using the last minute of V·̇O_2_ data from each of the five 4-min stages. Power output was then matched with a heart rate value by linear fit of the power output vs. heart rate relationship, using the average heart rate during the last minute of each 4-min stage. The VT_1_ power output was then converted to a rate of whole-body energy expenditure by linear fit of the whole-body energy expenditure vs. power output relationship. The whole-body rate of energy expenditure was calculated for each power output in the incremental assessment using the average V·̇O_2_ and V̇CO_2_ in the last minute of each of the five 4-min stages with a stoichiometric equation (Jeukendrup and Wallis 2005) (Eq. 2)

2 $${\text{Whole body rate of energy expenditure }}({\text{kcal}} \times \min ^{{ - 1}} ){\mkern 1mu} = {\mkern 1mu} 0.550{\mkern 1mu} \times {\mkern 1mu} \dot{V}CO_{2} {\mkern 1mu} + {\mkern 1mu} 4.471{\mkern 1mu} \times \dot{V}O_{2}$$

Where V·̇O_2_ and V·̇O_2_ are in L^.^min^−1^.

Using blood lactate data, the PRE and POST moderate-to-heavy intensity transitions were estimated using the LoglogLT method. The LoglogLT method models a blood lactate concentration vs. power output curve using two segments, and the intersection point of the two lines with the lowest residuals sum of squares is taken as the moderate-to-heavy intensity transition (Jamnick et al. 2018). The LoglogLT power output in the PRE and POST assessments were converted to heart rate, V·̇O_2_, and whole-body rate of energy expenditure values by linear fit of the relationships between these values and power output, as per above. As LoglogLT data produced essentially the same results as VT_1_, and the same inferences, only VT_1_ data are reported. The LoglogLT data can be found in Supplementary Figs. 1–2.

To quantify the proportion of prolonged exercise-induced changes in moderate-to-heavy intensity transition power output associated with changes in gross cycling efficiency and changes in rates of metabolic energy expenditure achieved at the moderate-to-heavy transition, rates of energy expenditure observed at VT_1_ and LoglogLT in the *POST* assessment were converted to a power output value using linear regression of the power output vs. energy expenditure relationship for each participant in the *PRE* assessment (denoted as POST_EE_PRE_Eff_). The POST_EE_PRE_Eff_ therefore indicates the power output that the rate of metabolic energy expenditure observed at the moderate-to-heavy transition in the POST assessment would have achieved with the level of gross cycling efficiency in the PRE assessment. Accordingly, the proportion of prolonged exercise-induced changes in VT_1_ and LoglogLT power output associated with changes in energetic efficiency and rates of metabolic energy expenditure achieved at the transition was calculated using the below equation (Eq. 3).

$${\text{Contribution of change in energetic efficiency to change in power output at the moderate}} - {\rm{to}} -{\text{heavy intensity transition }} = {\text{ POST}} - {\text{POST}}_{{{\rm{EE}}}} {\text{PRE}}_{{{\rm{Eff}}}}$$

3 $$\begin{gathered} {\text{Contribution of change in metabolic energy expenditureto change in power output at the moderate}} - {\text{to}} - {\text{heavy intensity transition}}\, \hfill \\ = \,{\text{POST}}_{{{\text{EE}}}} {\text{PRE}}_{{{\text{Eff}}}} {-}{\text{PRE}} \hfill \\ \end{gathered}$$

Equation 3 where PRE = power output at the moderate-to-heavy transition pre-prolonged exercise, POST = power output at the moderate-to-heavy transition post-prolonged exercise, and POST_EE_PRE_Eff_ = power output that would be produced in the PRE assessment using the rate of metabolic energy expenditure observed at the moderate-to-heavy transition in the POST assessment.

### Statistical analysis

Data are presented as mean ± standard deviation (SD), unless otherwise stated. Normality of data distributions were assessed using the Shapiro–Wilk test. The effect of prolonged exercise on moderate-to-heavy intensity transition power output, heart rate, V·̇O_2_, and rate of energy expenditure was assessed using paired t-tests (or the non-parametric equivalent Wilcoxon test). Relationships between PRE to POST changes in moderate-to-heavy transition power output and PFO, V·̇O_2_peak, the moderate-to-heavy intensity transition in the PRE assessment, sweat loss, and dehydration were assessed using Pearson’s (r) or Spearman’s rank-order (r_s_) correlation coefficients, depending on normality, and expressed with 95% confidence intervals. Changes in heart rate, whole-body energy expenditure, V·̇O_2_, and respiratory exchange ratio (RER) over-time during the two-hour constant work-rate phase were analysed using repeated measures one-way analyses of variance. Whole-body fat oxidation rates during the first three stages of the PRE and POST incremental tests were compared using a mixed model analysis of variance due to missing data-points. Variance was located post-hoc using Holm-Bonferroni stepwise correction. Analyses were performed in GraphPad Prism Version 9.3.1. Significance was inferred when *P* ≤ 0.05.

## Results

### Constant work-rate phase

**Fig. 2:**
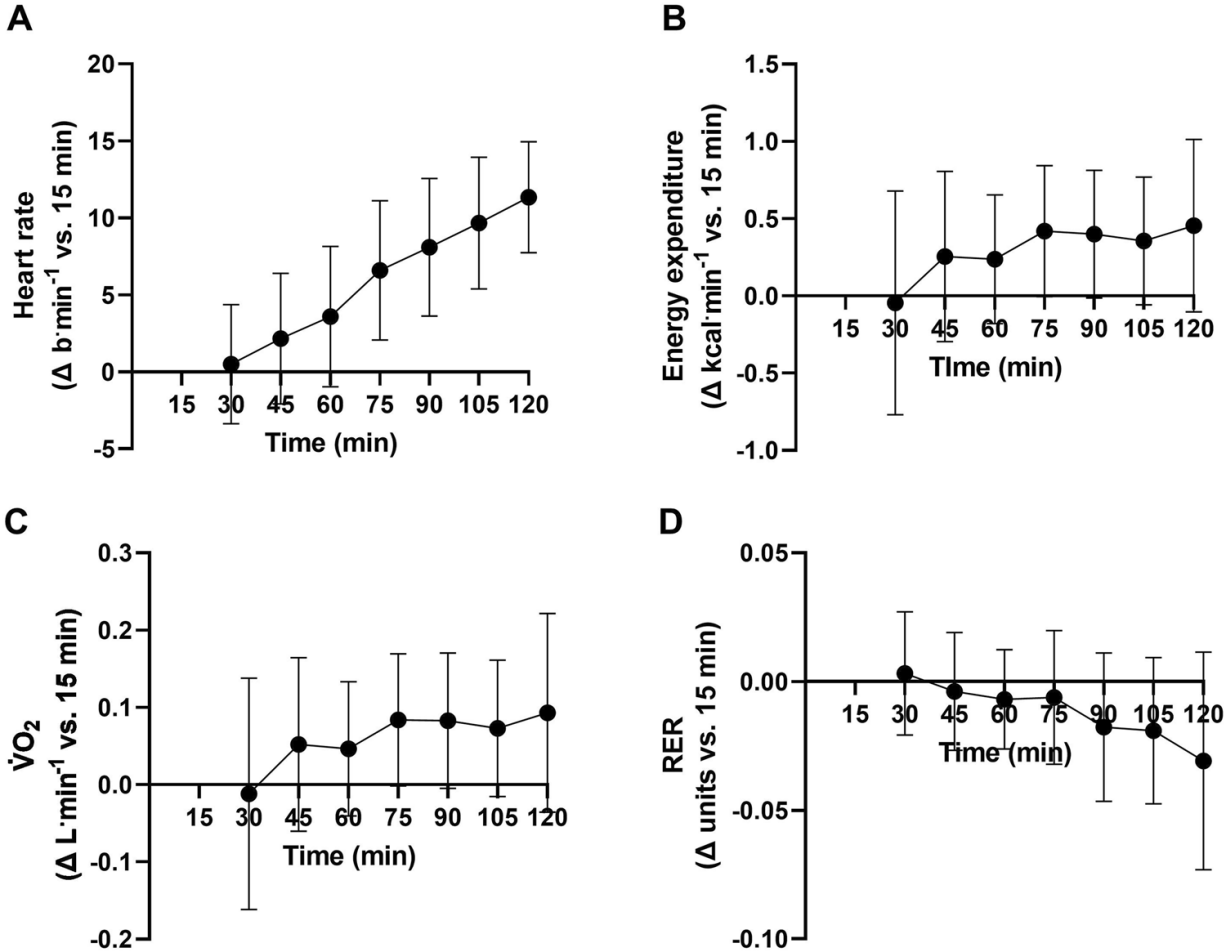
Changes in **a** heart rate, **b** whole-body energy expenditure, **c** V·̇O_2_, and **d** respiratory exchange ratio (RER) vs. the 15 min timepoint during the two-hour constant work-rate phase

The estimated power output at VT_1_ in the initial assessment was 216 ± 45 W. Consequently, the two-hour constant work-rate phase in the experimental trial was completed at 194 ± 41 W. From 15 to 120 min of the two-hour constant work-rate phase, heart rate significantly increased (*P* < 0.0001, 8.2 ± 2.7%, Fig. 2a). Rates of whole-body energy expenditure (*P* = 0.07, 3.4 ± 4.3%, Fig. 2b) and V·̇O_2_ (*P* = 0.08, 3.4 ± 4.9%, Fig. 2c) did not increase during the two-hour constant work-rate phase, although both effects approached significance. The RER did not change during the two-hour constant work-rate phase (*P* = 0.61, Fig. 2d).

### Moderate-to-heavy intensity transition

**Fig. 3:**
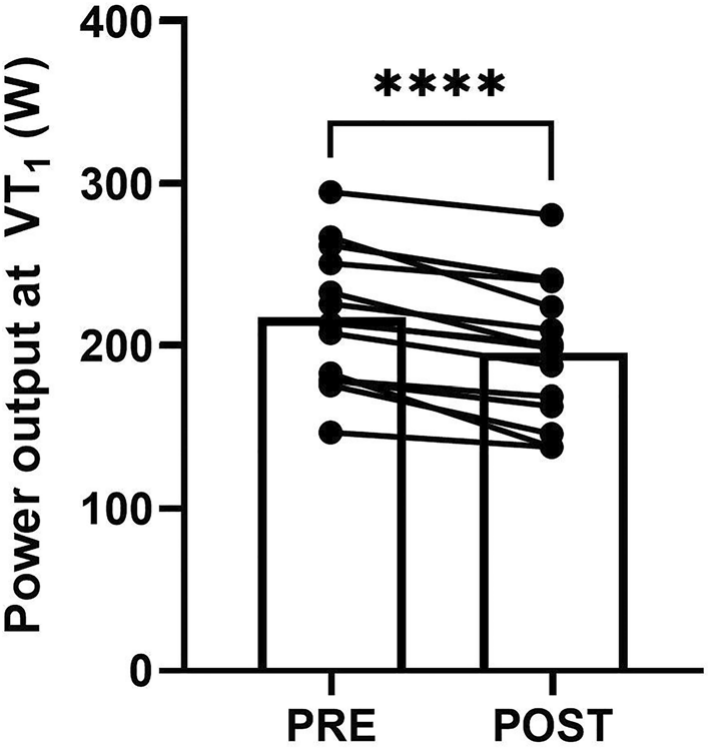
Power output at the moderate-to-heavy intensity transition before (PRE) and after (POST) prolonged cycling as determined by the first ventilatory threshold (VT_1_). Bars indicate mean values and lines indicate individual responses. **** denotes *P* ≤ 0.0001

The power output at VT_1_ significantly decreased from PRE to POST (217 ± 42 W vs. 196 ± 42 W, ∆ − 21 ± 12 W, ∆ − 10.0 ± 5.8%, *P* < 0.0001, Fig. 3). The magnitude of PRE to POST change in VT_1_ power output was not significantly associated with PFO, V·̇O_2_peak, prolonged exercise-induced sweat loss, or prolonged exercise-induced dehydration (Table 1). However, the magnitude of the reduction in VT_1_ power output from PRE to POST was be related to the VT_1_ expressed as %V·̇O_2_peak in the PRE assessment.

**Table 1:** Bivariate associations between durability of the moderate-to-heavy transition (Δ VT_1_ expressed in absolute units, W, and as a percentage of PRE values) and outcome measures in this study. Data are reported as Spearman’s rank-order (r_s_) correlation coefficients (95% confidence intervals), with accompanying *P* values

	Δ VT_1_ (W)	Δ VT_1_ (%)
PFO (g^.^min^−1^)	r_s_ = − 0.13 (− 0.63, 0.44) *P* = 0.66	r_s_ = 0.09 (− 0.47, 0.61) *P* = 0.75
∆ FO step 1 (g^.^min^−1^)	r_s_ = − 0.14 (− 0.63, 0.43) *P* = 0.63	r_s_ = 0.17 (− 0.43, 0.67) *P* = 0.58
∆ FO step 2 (g^.^min^−1^)	r_s_ = − 0.24 (− 0.69, 0.45) *P* = 0.40	r_s_ = 0.22 (− 0.39, 0.70) *P* = 0.46
∆ FO step 3 (g^.^min^−1^)	r_s_ = − 0.18 (− 0.67, 0.43) *P* = 0.57	r_s_ = 0.20 (− 0.44, 0.71) *P* = 0.42
V·̇O_2_peak (mL^.^kg^−1.^min^−1^)	r_s_ = − 0.23 (− 0.69, 0.36) *P* = 0.43	r_s_ = 0.18 (− 0.41, 0.66) *P* = 0.54
V·̇O_2_peak (L^.^min^−1^)	r_s_ = − 0.35 (− 0.75, 0.24) *P* = 0.22	r_s_ = 0.03 (− 0.52, 0.56) *P* = 0.92
PRE VT_1_ (%V·̇O_2_peak)	**r**_**s**_** = 0.54** **(− 0.00, 0.84)** ***P* = 0.05**	r_s_ = 0.52 (− 0.03, 0.83) *P* = 0.06
Sweat loss (L)	r_s_ = − 0.17 (− 0.71, 0.49) *P* = 0.61	r_s_ = 0.23 (− 0.45, 0.74) *P* = 0.49
Dehydration (% of BM)	r_s_ = 0.43 (− 0.25, 0.82) *P* = 0.19	r_s_ = 0.50 (− 0.16, 0.85) *P* = 0.19

∆ FO step 1–3 = change in whole-body fat oxidation rate from PRE to POST in step 1–3, *BM* body mass, *PFO* peak fat oxidation rate observed in the initial assessment, V·̇O_2_peak = peak oxygen uptake, and Δ VT_1_ = prolonged exercise-induced change in the first ventilatory threshold power output. Significant relationships (*P* ≤ 0.05) are highlighted in **bold**

**Fig. 4:**
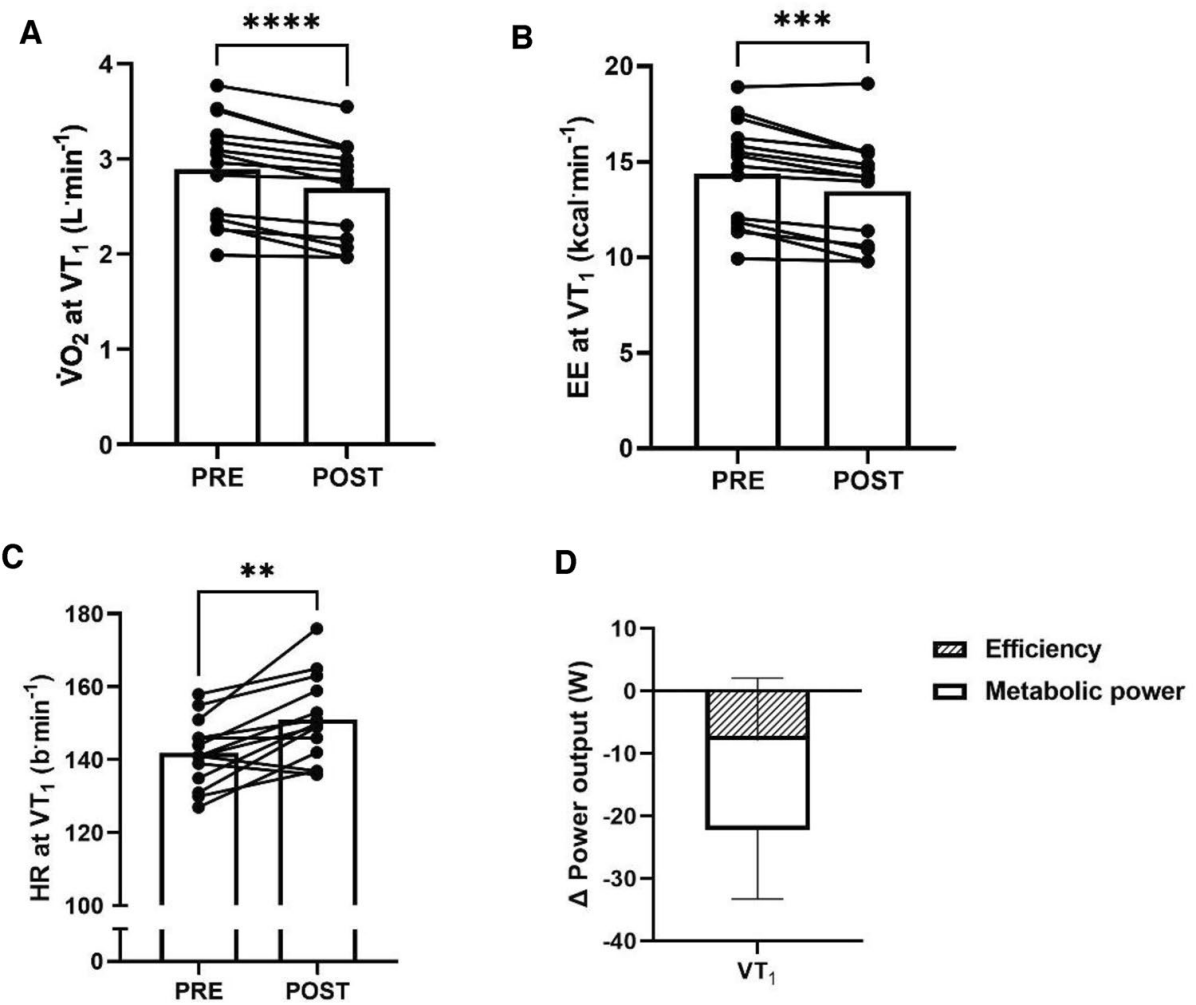
The **a** rate of oxygen consumption (V·̇O_2_), **b** energy expenditure (EE), and **c** heart rate (HR) at the first ventilatory threshold (VT_1_) before (PRE) and after (POST) prolonged exercise. The contributions to prolonged exercise-induced changes in VT_1_ power output made by loss of efficiency and metabolic energy expenditure at the transition is shown in **d**. Bars indicate mean values and lines indicate individual responses. *** denotes *P* ≤ 0.001, **** denotes *P* ≤ 0.0001

The V·̇O_2_ at VT_1_ significantly decreased from PRE to POST (2.89 ± 0.55 vs. 2.69 ± 0.51 L^.^min^−1^, 68 ± 7 vs. 63 ± 8% of V·̇O_2_peak, *P* < 0.0001, Fig. 4a). The rate of energy expenditure at VT_1_ significantly decreased from PRE to POST (14.4 ± 2.7 vs. 13.5 ± 2.7 kcal^.^min^−1^, *P* = 0.0002, Fig. 4b). The heart rate at VT_1_ significantly increased from PRE to POST (142 ± 9 vs. 151 ± 12 beats^.^min^−1^, *P* = 0.001, Fig. 4c). The exercise-induced decrease in power output at VT_1_ was attributable to decreased energetic efficiency (-8 ± 10 W) and rates of metabolic energy expenditure at the transition (− 14 ± 11 W, Fig. 4d). The relative contribution made by decreased efficiency and rates of metabolic energy expenditure to the decrease in power output at VT_1_ was not significantly different (*P* = 0.18).

**Fig. 5:**
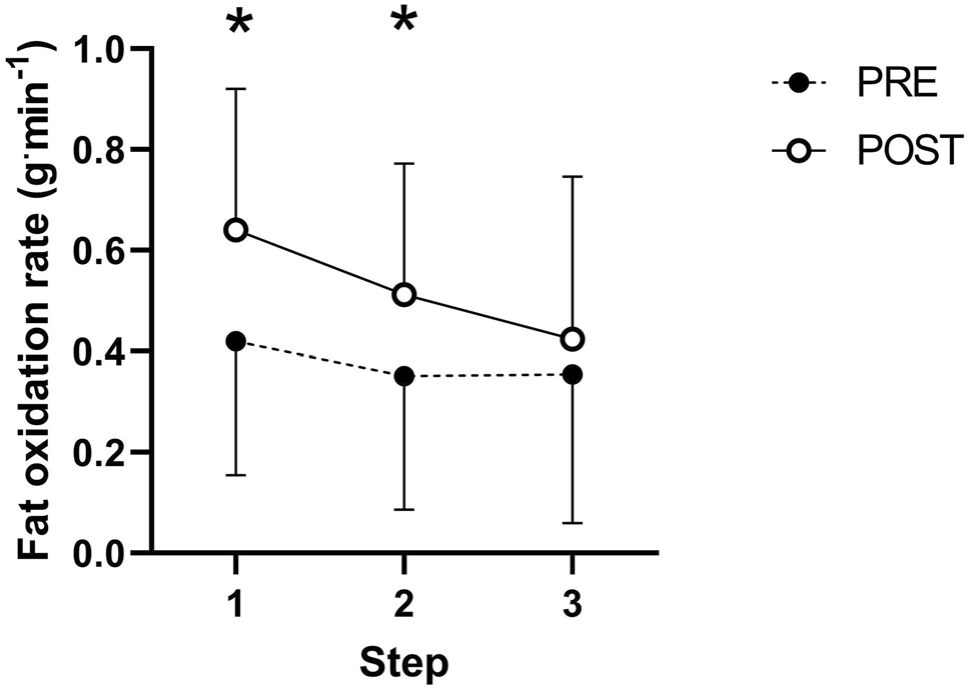
The whole-body fat oxidation rate (g^.^min^−1^) in steps 1, 2, and 3 of the incremental exercise tests before (PRE) and after (POST) the two-hour constant work-rate phase in the prolonged trial. Circles indicate means and error bars indicate standard deviations. Data is plotted for the first three steps as in some instances the respiratory exchange ratio approached or exceeded 1.0 in the fourth and fifth steps if the POST assessment. * denotes *P* ≤ 0.05 in PRE vs. POST

There was an effect of prolonged exercise (*P* = 0.04), and prolonged exercise by intensity interaction (*P* = 0.002), on whole-body fat oxidation rates during the PRE and POST assessments. Specifically, whole-body fat oxidation rates were greater in the POST vs. PRE assessment during the first and second steps (Fig. 5). The loss of power output at the moderate-to-heavy intensity transition associated with decreased energetic efficiency was significantly associated with changes in whole-body fat oxidation rates from PRE to POST, but these relationships were not present for the loss of power output associated with rates of metabolic energy expenditure at the transition (Table 2, Supplementary Fig. 3).

**Table 2:** Bivariate associations between PRE to POST changes in whole-body fat oxidation rates in steps 1, 2, and 3 of the incremental exercise tests with the contributions to prolonged exercise-induced changes in moderate-to-heavy intensity transition power output made by loss of efficiency and metabolic energy expenditure at the transition. Data are reported as Pearson’s product-moment (r) correlation coefficients (95% confidence intervals), with accompanying *P* values

	∆ FO step 1 (g^.^min^−1^)	∆ FO step 2 (g^.^min^−1^)	∆ FO step 3 (g^.^min^−1^)
∆ Efficiency (VT_1_, W)	**r = − 0.72** **(− 0.90, − 0.30)** ***P* = 0.004**	**r = −0.74 (− 0.91, − 0.34)* P* = 0.003**	**r = − 0.57 (− 0.85, − 0.02)* P* = 0.04**
∆ Metabolic EE (VT_1_, W)	r = 0.36 (− 0.21, 0.75) *P* = 0.20	r = 0.31 (− 0.26, 0.72) *P* = 0.28	r = 0.15 (− 0.44, 0.65)* P* = 0.63

∆ Efficiency = contribution made by changes in efficiency to prolonged-exercise-induced changes in the moderate-to-heavy intensity transition, ∆ FO step 1–3 = change in whole-body fat oxidation rate from PRE to POST in step 1–3, ∆ Metabolic EE = contribution made by changes in metabolic energy expenditure at the transition to prolonged-exercise-induced changes in the moderate-to-heavy intensity transition. Significant relationships (*P* ≤ 0.05) are highlighted in **bold**

## Discussion

The aim of this study was to determine the effects of prolonged moderate-intensity cycling on the moderate-to-heavy intensity transition power output and heart rate. Our primary observations were that: (i) the power output at the moderate-to-heavy intensity transition significantly decreased following prolonged cycling, (ii) this loss of power output was attributable to reduced gross cycling energetic efficiency *and* reduced rates of metabolic energy expenditure at the transition, and (iii) the heart rate associated with the moderate-to-heavy intensity transition increased following prolonged cycling. These data have implications for athlete profiling, training load monitoring, and training programming, and indicate that the ‘durability’ of the moderate-to-heavy intensity transition warrants attention at the individual level.

The observed reduction in moderate-to-heavy intensity transition power output following prolonged exercise was partially accounted for by reduced energetic efficiency, or the translation of metabolic energy expenditure to mechanical power output (Fig. 4d). Loss of efficiency was also demonstrated during the two-hour constant work-rate phase, as whole-body rates of energy expenditure (*P* = 0.07, Fig. 2b) and V·̇O_2_ (*P* = 0.08, Fig. 2b) at the fixed power output increased over-time, albeit not significantly. These observations are in line with previous work demonstrating decreased gross cycling efficiency following prolonged exercise (Passfield and Doust 2000; Hopker et al. 2017). Mechanistically, the rising energy and V·̇O_2_ cost of given power outputs following prolonged exercise may reflect progressive loss of skeletal muscle contractile efficiency due to increasing recruitment of less efficient type II muscle fibres (Jones et al. 2011). The increased V·̇O_2_ cost of producing a fixed power output would have been exacerbated beyond the loss of energetic efficiency by the increased fat oxidation (Fig. 5), as fat oxidation requires more V·̇O_2_ per unit of energy produced than carbohydrate oxidation (Frayn 1983).

The remainder of the reduction in moderate-to-heavy intensity transition power output was accounted for by decreased rates of metabolic energy expenditure at the transition (Fig. 4d). This is demonstrated by the reduction in rates of energy expenditure as well as V·̇O_2_ at the moderate-to-heavy intensity transition from PRE to POST (Fig. 4ab). Plausibly, the observed reduction in energy expenditure at the moderate-to-heavy intensity transition with prolonged exercise may also be at least partially attributable to decreased endogenous carbohydrate availability. Specifically, localised glycogen depletion in the intramyofibrillar compartment has been linked to impaired excitation–contraction coupling, manifesting as reduced Ca^2+^ release from the sarcoplasmic reticulum under neural innervation (Ørtenblad et al. 2013). Therefore, intramyofibrillar glycogen depletion during the prolonged exercise of the current study may plausibly have diminished the function of individual working muscle fibres. Evidence for depletion of endogenous carbohydrate availability is provided by the observed increase in whole-body fat oxidation rates from PRE to POST (Fig. 5), given the autoregulatory nature of muscle glycogen metabolism (Hargreaves et al. 1995). In turn, impaired contractile activity of specific muscle fibres due to intramyofibrillar glycogen depletion may have increased the metabolic burden that a given power output placed on the smaller number of active, fully functional fibres. More specifically, greater burden may have been placed on less oxidative type IIAB and IIB later in the prolonged exercise bout, as evidenced by prior work on fibre type-specific glycogen depletion patterns during prolonged exercise (Vøllestad et al. 1984). These proposed effects of glycogen depletion on the moderate-to-heavy transition may have been exacerbated by the exercise of the present study being conducted after an overnight fast and without carbohydrate intake during exercise, and may plausibly therefore be lessened in training and competition scenarios in which exercise is performed postprandially and with carbohydrate feeding. However, as muscle glycogen depletion, and more specifically compartmental muscle glycogen depletion, was not measured in this study, this mechanism remains speculative and could be interrogated in future work. Additionally, the importance of glycogen availability for durability of the moderate-to-heavy transition could be further explored through repetition of the present protocol with experimental manipulation of pre-exercise glycogen availability through exercise and/or nutrition interventions.

Interestingly, the contribution made by decreased efficiency, but not decreased rates of metabolic energy expenditure at the transition, to prolonged exercise-induced changes in the moderate-to-heavy intensity transition power output was related to the magnitude of PRE to POST changes in whole-body fat oxidation rates; that is, those for whom decreases in energetic efficiency were large exhibited the largest PRE to POST increases in whole-body fat oxidation rates (Table 2). Larger increases in fat oxidation following prolonged exercise may reflect greater muscle glycogen depletion (Hargreaves et al. 1995), and glycogen depletion may as discussed negatively impact contractile function and in turn efficiency at the level of the muscle fibre (Ørtenblad et al. 2013). These data may therefore indirectly support that the loss of energetic efficiency with prolonged exercise was at least partially attributable to the degree of glycogen depletion. However, these data are associational and muscle glycogen was not measured in the present study, and so this mechanism should be interrogated directly in future work.

In contrast, the reduction in moderate-to-heavy intensity transition power output was not significantly associated with PFO (Table 1). The PFO is a marker of an individual’s capacity for fat oxidation during exercise (Maunder et al. 2018), meaning that having a greater capacity to oxidise fatty acids in a fresh state during exercise was not related to ‘durability’ of the moderate-to-heavy intensity transition. If muscle glycogen depletion was the primary mechanism behind the observed prolonged exercise-induced reduction in moderate-to-heavy intensity transition power output, one might have predicted that possessing a greater capacity to oxidise fatty acids during exercise would have mitigated this decline. The absence of a relationship between PFO and the durability of the moderate-to-heavy intensity transition therefore appears to counter this proposed mechanism. However, PFO may not completely reflect the degree of muscle glycogen depletion induced by the prolonged exercise, and therefore to test this hypothesis future work may consider replicating the design of the present study, but with measurements of muscle glycogen content.

As the power output at the moderate-to-heavy intensity transition decreased following acute prolonged exercise, these data suggest that using a well-rested assessment of power output at the moderate-to-heavy intensity transition for programming prolonged exercise risks inadvertent drift from the moderate into the heavy intensity domain. This may have implications for training prescription; specifically, drift into the heavy domain may extend the recovery required after sessions intended to be of moderate intensity and therefore low physiological stress (Seiler et al. 2007; Stanley et al. 2013). Similarly, training load models may need to consider accounting for the durability of intensity domain transitions to better quantify training load.

During the two-hour constant work-rate phase, heart rate increased by 8.2 ± 2.7% from 15 to 120 min (Fig. 2a). This may have been related to increases in core temperature and therefore cutaneous blood flow, progressive dehydration and therefore reduced stroke volume (Coyle and Gonzalez-Alonso 2001), as well as the increased metabolic demand of the fixed work rate (Fig. 2b). More importantly, and in contrast to our hypothesis, it was observed that the heart rate associated with VT_1_ significantly increased from PRE to POST (6.3 ± 5.8%, Fig. 4c). These data demonstrate that the cardiovascular drift that occurred with acute prolonged exercise was proportionally larger than the downward drift in the power output associated with the moderate-to-heavy intensity transition, and therefore that the heart rate associated with the moderate-to-heavy intensity transition increases over time during acute prolonged exercise. These results therefore suggest use of well-rested assessments of the heart rate at the moderate-to-heavy intensity transition to prescribe prolonged exercise may risk ‘undertraining’, or downward drift within the moderate-intensity domain over time.

In the present study there was inter-individual variation in the degree of reduction in moderate-to-heavy intensity transition power output following prolonged exercise, with the reduction in VT_1_ power output ranging from ~ 9–44 W (Fig. 3). This suggests that the durability of the moderate-to-heavy intensity transition is not a uniform characteristic between-athletes, and thus that profiling the effects of prolonged exercise at the individual level may be useful for capturing an endurance athlete’s physiological profile. Moderate strength relationships were observed between the initial VT_1_ power output, expressed as %V·̇O_2_peak, and the durability of the moderate-to-heavy intensity transition, although this relationship was not present when the moderate-to-heavy intensity transition was estimated using blood lactate data (Table 1). Future work may consider exploring the implications of this characteristic for endurance performance, and also the training-related and physiological characteristics that differentiate athletes with high vs. low durability.

In conclusion, the present investigation demonstrated prolonged moderate-intensity cycling significantly reduced the power output observed at the moderate-to-heavy intensity transition. This reduction was associated with decreased gross cycling efficiency and rates of metabolic energy expenditure at the transition. The heart rate associated with this transition increased following prolonged exercise. Therefore, it may be important for endurance athletes to understand how intensity transitions are affected by prolonged exercise at an individual level to refine physiological profiling, training prescription, and load monitoring.

## Supplementary Information

Below is the link to the electronic supplementary material.

Supplementary file1 (JPG 121 KB)

Supplementary file2 (JPG 77 KB)

Supplementary file3 (JPG 75 KB)

Supplementary file4 (JPG 135 KB)

Supplementary file5 (JPG 52 KB)

Supplementary file6 (PDF 61 KB)

## Data Availability

Data are available from the corresponding author upon reasonable request.

## Abbreviations

EE: Energy expenditure
HR: Heart rate
PFO: Peak fat oxidation
$\dot{V}$CO_2_: Rate of carbon dioxide production
$\dot{V}_{E}$: Rate of ventilation
$\dot{V}$O_2_: Rate of oxygen consumption
$\dot{V}$O_2_peak: Peak rate of oxygen consumption
VT_1_: First ventilatory threshold

## Code availability

None used.

## References

[CR1] Burnley M, Jones AM (2018). Power–duration relationship: Physiology, fatigue, and the limits of human performance. Eur J Sport Sci.

[CR2] Clark IE, Vanhatalo A, Bailey SJ (2018). Effects of two hours of heavy-intensity exercise on the power–duration relationship. Med Sci Sports Exerc.

[CR3] Clark IE, Vanhatalo A, Thompson C (2019). Dynamics of the power-duration relationship during prolonged endurance exercise and influence of carbohydrate ingestion. J Appl Physiol.

[CR4] Clark IE, Vanhatalo A, Thompson C (2019). Changes in the power-duration relationship following prolonged exercise: estimation using conventional and all-out protocols and relationship with muscle glycogen. Am J Physiol - Regul Integr Comp Physiol.

[CR5] Coyle EF, Gonzalez-Alonso J (2001). Cardiovascular drift during prolonged exercise: new perspectives. Exerc Sport Sci Rev.

[CR6] Frayn KN (1983). Calculation of substrate oxidation rates in vivo from gaseous exchange. J Appl Physiol.

[CR7] Hargreaves M, McConell G, Proietto J (1995). Influence of muscle glycogen on glycogenolysis and glucose uptake during exercise in humans. J Appl Physiol.

[CR8] Hopker JG, O’Grady C, Pageaux B (2017). Prolonged constant load cycling exercise is associated with reduced gross efficiency and increased muscle oxygen uptake. Scand J Med Sci Sport.

[CR9] Jamnick NA, Botella J, Pyne DB, Bishop DJ (2018). Manipulating graded exercise test variables affects the validity of the lactate threshold and VO2peak. PLoS ONE.

[CR10] Jeukendrup AE, Wallis GA (2005). Measurement of substrate oxidation during exercise by means of gas exchange measurements. Int J Sports Med.

[CR11] Jones AM, Grassi B, Christensen PM (2011). Slow component of VO2 kinetics: mechanistic bases and practical applications. Med Sci Sports Exerc.

[CR12] Jones AM, Burnley M, Black MI (2019). The maximal metabolic steady state redefining the gold standard. Physiol Rep.

[CR13] Maunder E, Plews DJ, Kilding AE (2018). Contextualising maximal fat oxidation during exercise: determinants and normative values. Front Physiol.

[CR14] Maunder E, Seiler S, Mildenhall MJ (2021). The importance of ‘durability’ in the physiological profiling of endurance athletes. Sports Med.

[CR15] Maunder E, Plews DJ, Wallis GA (2022). Peak fat oxidation is positively associated with vastus lateralis CD36 content, fed-state exercise fat oxidation, and endurance performance in trained males. Eur J Appl Physiol.

[CR16] Ørtenblad N, Westerblad H, Nielsen J (2013). Muscle glycogen stores and fatigue. J Physiol.

[CR17] Passfield L, Doust JH (2000). Changes in cycling efficiency and performance after endurance exercise. Med Sci Sports Exerc.

[CR18] Seiler S, Haugen O, Kuffel E (2007). Autonomic recovery after exercise in trained athletes: Intensity and duration effects. Med Sci Sports Exerc.

[CR19] Stanley J, Peake JM, Buchheit M (2013). Cardiac parasympathetic reactivation following exercise: implications for training prescription. Sports Med.

[CR20] Vøllestad NK, Vaage O, Hermansen L (1984). Muscle glycogen depletion patterns in type I and subgroups of type II fibres during prolonged severe exercise in man. Acta Physiol Scand.

