# Supplementary figures and images for: Prolonged cycling reduces power output at the moderate-to-heavy intensity transition
Source: Eur J Appl Physiol. 2022 Sep 20;122(12):2673–82. doi: 10.1007/s00421-022-05036-9 (PMC9488873; doi:10.1007/s00421-022-05036-9)

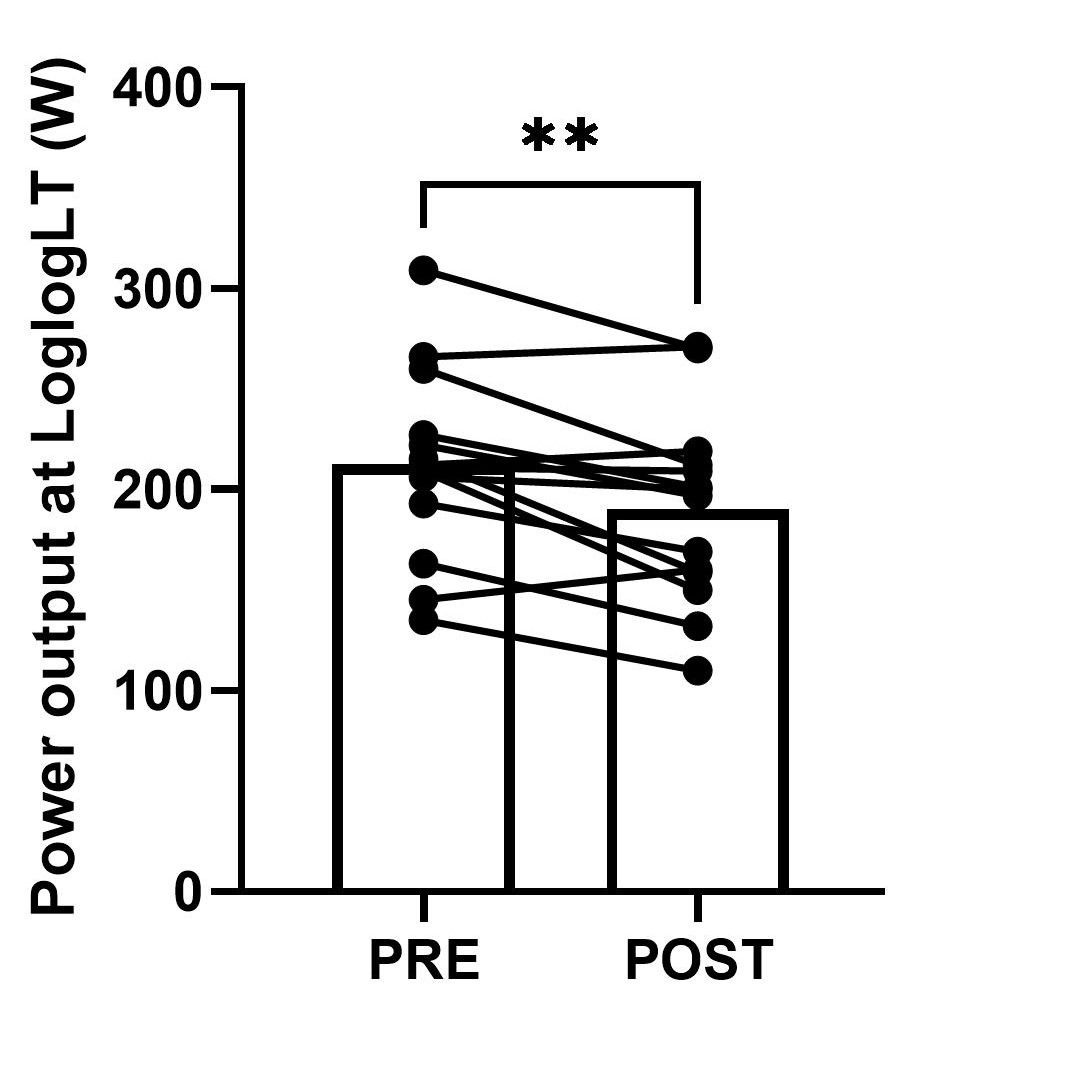

Supplement: Supplementary file 1 — Supplementary file1 (JPG 121 KB) [file 421_2022_5036_MOESM1_ESM.jpg]

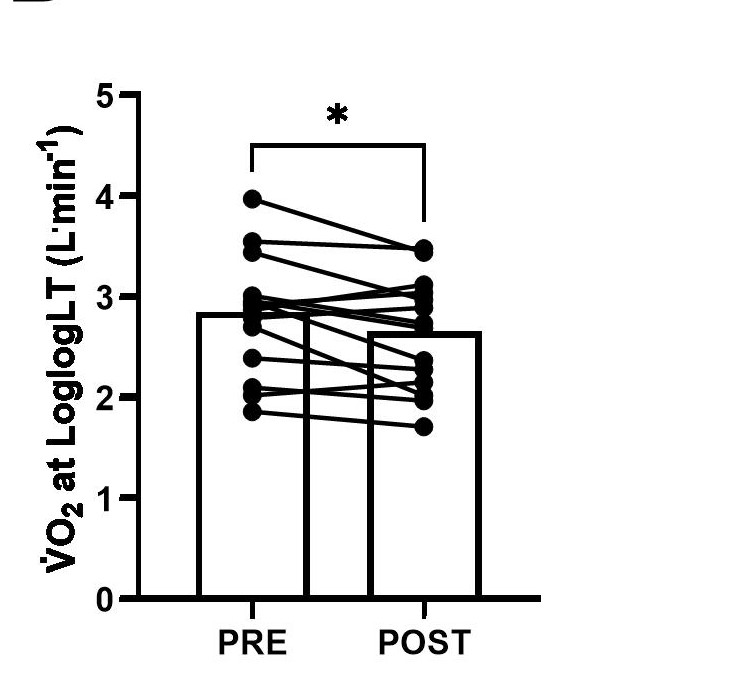

Supplement: Supplementary file 2 — Supplementary file2 (JPG 77 KB) [file 421_2022_5036_MOESM2_ESM.jpg]

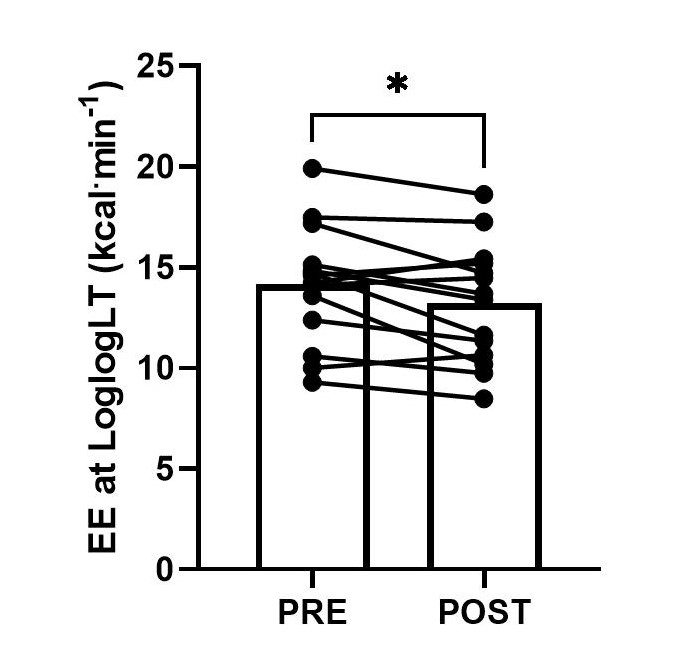

Supplement: Supplementary file 3 — Supplementary file3 (JPG 75 KB) [file 421_2022_5036_MOESM3_ESM.jpg]

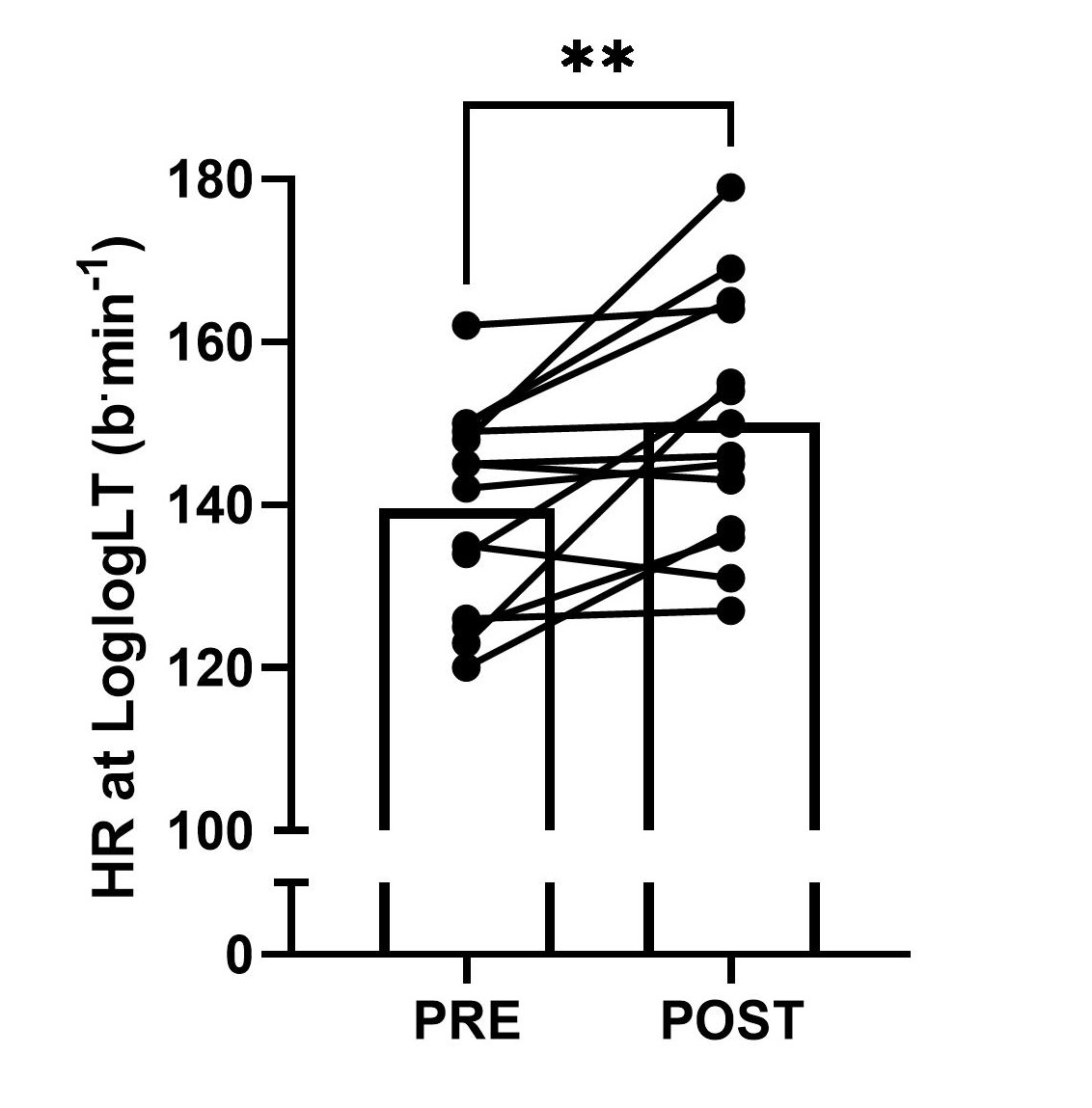

Supplement: Supplementary file 4 — Supplementary file4 (JPG 135 KB) [file 421_2022_5036_MOESM4_ESM.jpg]

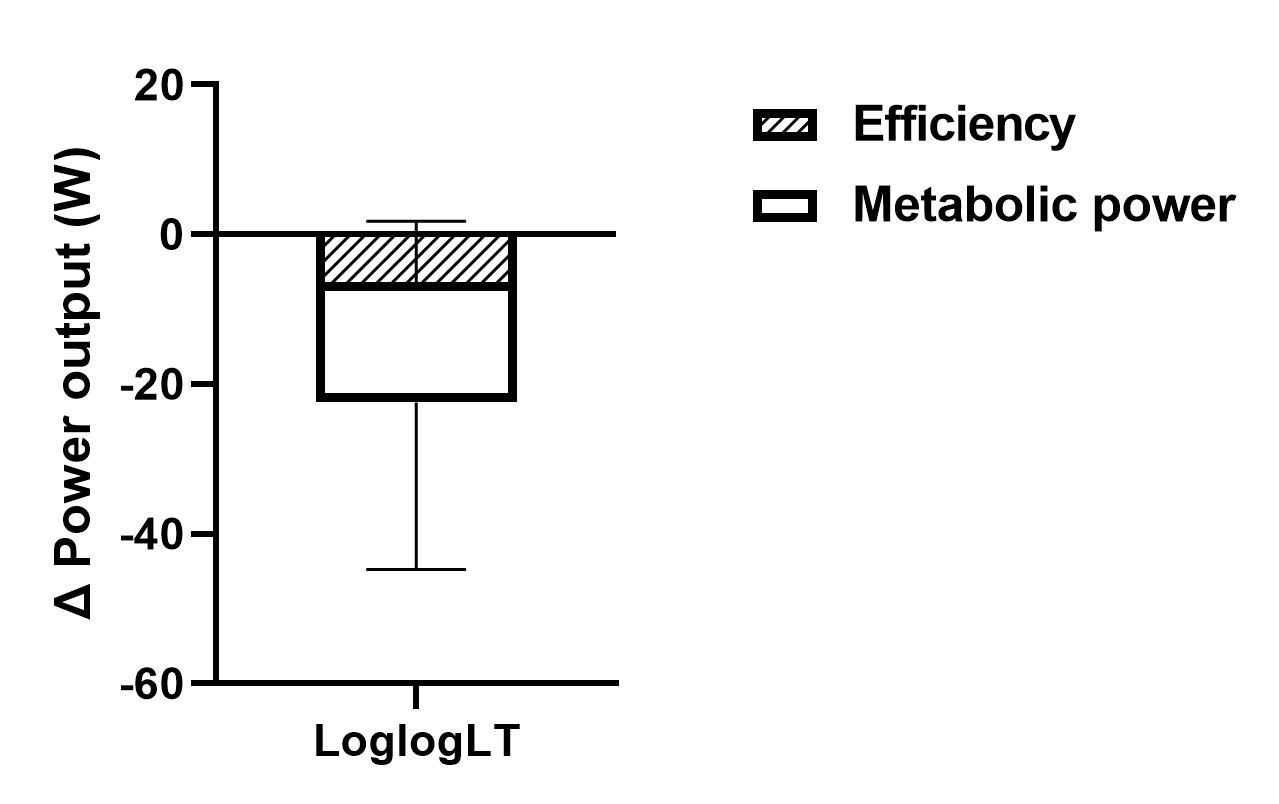

Supplement: Supplementary file 5 — Supplementary file5 (JPG 52 KB) [file 421_2022_5036_MOESM5_ESM.jpg]

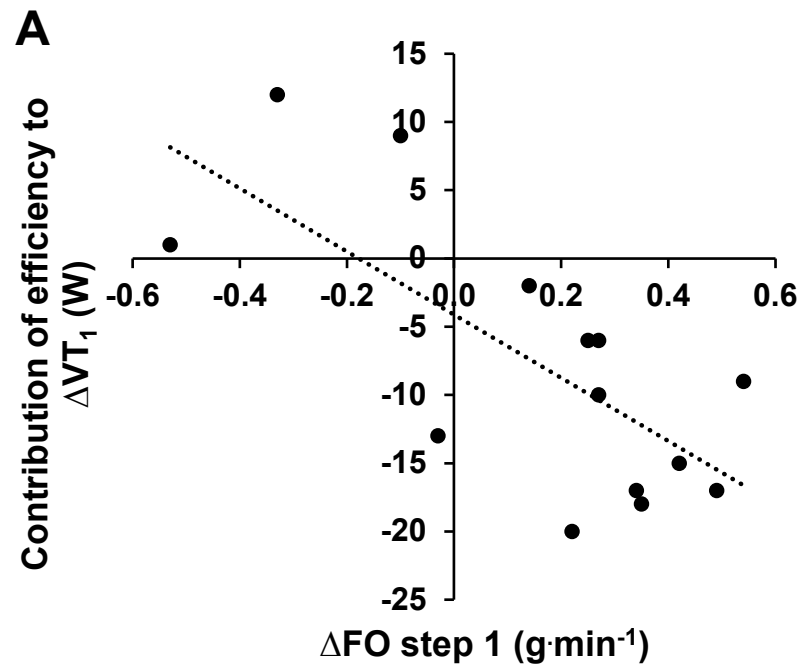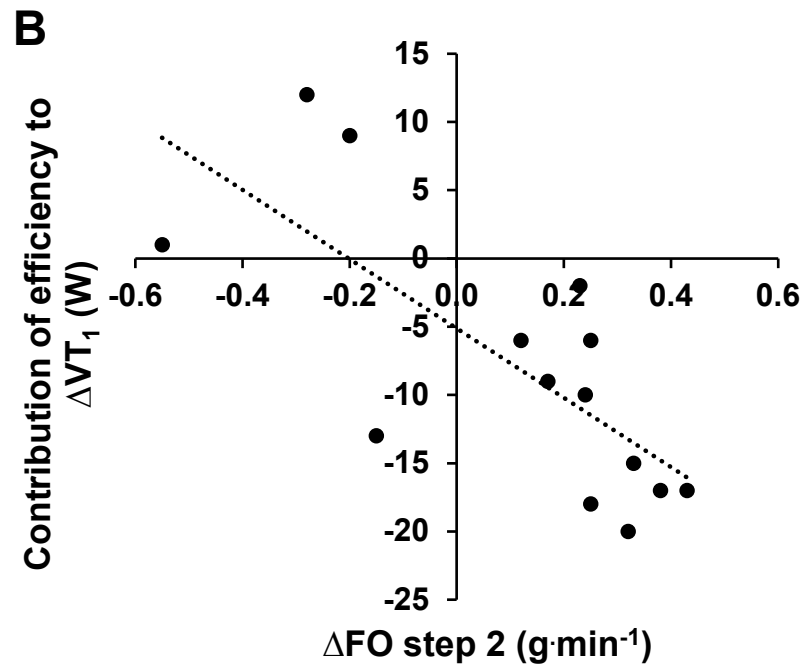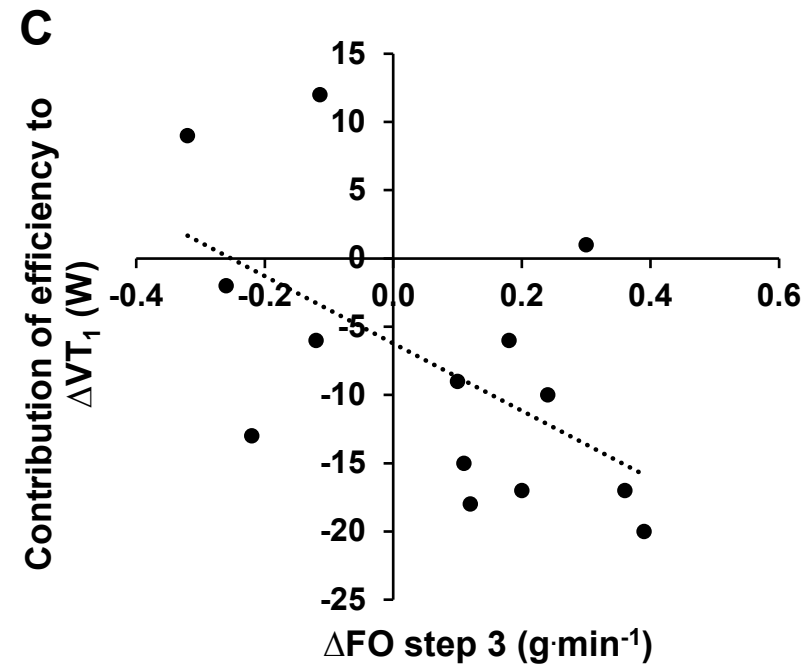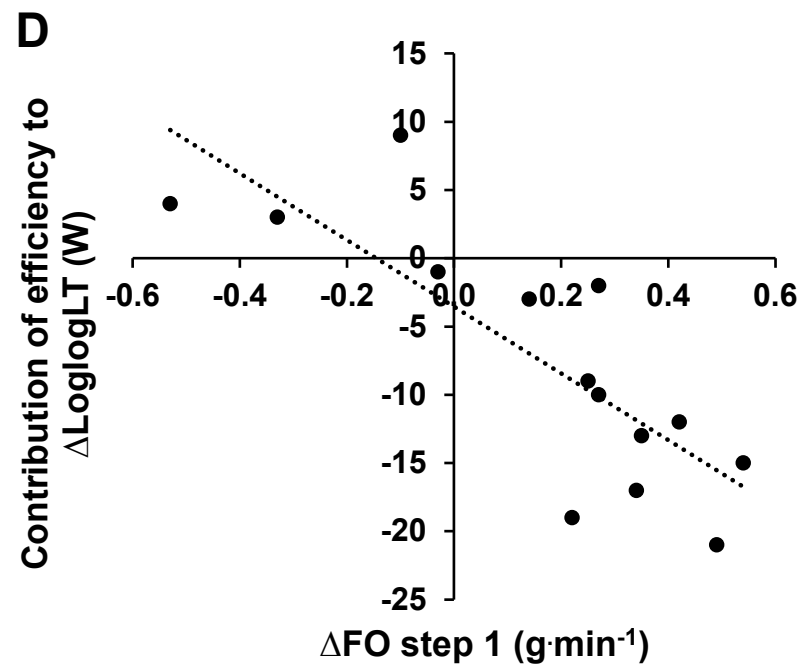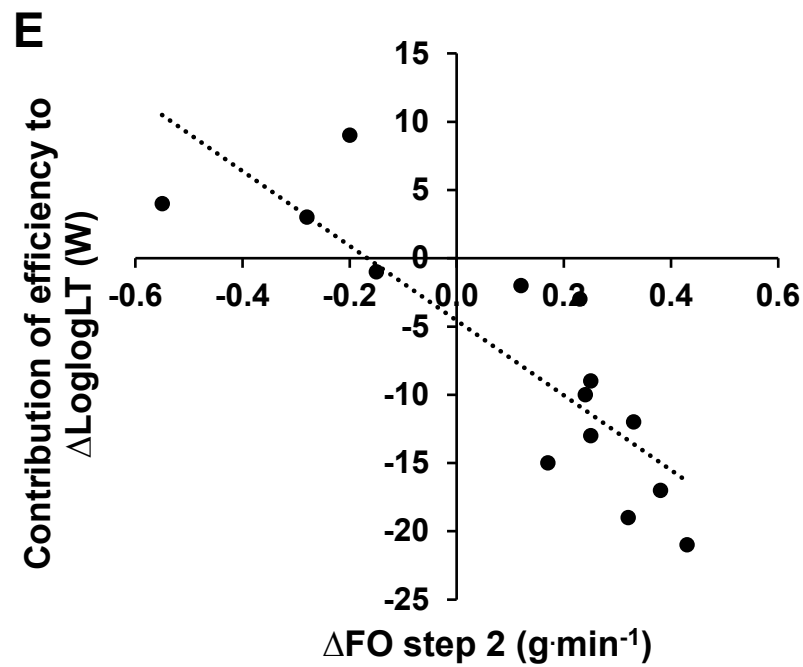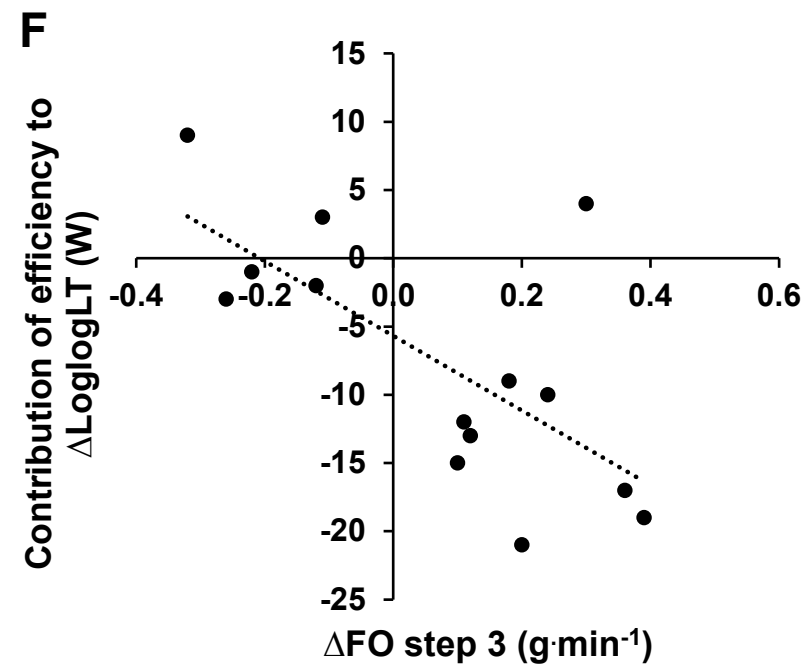

Supplement: Supplementary file 6 — Supplementary file6 (PDF 61 KB) [file 421_2022_5036_MOESM6_ESM.pdf]
